# Support of Unrelated Stem Cell Donor Searches by Donor Center-Initiated HLA Typing of Potentially Matching Donors

**DOI:** 10.1371/journal.pone.0020268

**Published:** 2011-05-20

**Authors:** Alexander H. Schmidt, Ute V. Solloch, Daniel Baier, Alois Grathwohl, Jan Hofmann, Julia Pingel, Andrea Stahr, Gerhard Ehninger

**Affiliations:** 1 DKMS German Bone Marrow Donor Center, Tübingen, Germany; 2 Internal Medicine I, University Hospital Carl Gustav Carus, Dresden, Germany; Karolinska Institutet, Sweden

## Abstract

Large registries of potential unrelated stem cell donors have been established in order to enable stem cell transplantation for patients without HLA-identical related donors. Donor search is complicated by the fact that the stored HLA information of many registered donors is incomplete. We carried out a project that was aimed to improve chances of patients with ongoing donor searches to find an HLA-matched unrelated donor. For that purpose, we carried out additional donor center-initiated HLA-DRB1 typing of donors who were only typed for the HLA loci A and B so far and were potential matches for patients in need of a stem cell transplant. In total, 8,861 donors were contacted for donor center-initiated HLA-DRB1 typing within 1,089 donor searches. 12 of these donors have donated stem cells so far, 8 thereof for their respective target patients. We conclude that chances of patients with ongoing donor searches to find an HLA-matched unrelated donor can indeed be improved by donor-center initiated typing that is carried out in addition to the standard donor search process. Our results also raise questions regarding the appropriate use of incompletely typed donors within unrelated donor searches.

## Introduction

Allogeneic hematopoietic stem cell transplantation is a well-established and increasingly used therapy for hematological malignancies and other severe diseases of the blood [1], [2]. In the absence of HLA-identical related donors, HLA-matched unrelated volunteers donate hematopoietic stem cells either from bone marrow or peripheral blood. For this purpose, national registries have been built up since the 1970s. These registries administer anonymous HLA data of registered donors and manage national and international donor searches. Donor centers, on the other hand, are responsible for education, recruitment, and HLA typing of potential stem cell donors. Donor centers also communicate with registered donors during the various steps of the donor search process. Currently, about 17.0 million potential stem cell donors are registered worldwide [3].

Full HLA typing, i.e., high-resolution typing of at least the HLA loci A, B, C and DRB1, of newly registered potential stem cell donors is advantageous as it reflects the current standard of donor-recipient matching [4]-[6]. Due to cost and capacity reasons, however, this typing strategy became possible only some years ago. Therefore, the worldwide file of potential stem cell donors that has grown for decades consists mainly of incompletely typed donors. The existence of incompletely typed donors on the worldwide donor file obviously complicates unrelated donor searches and makes it possible that a fully matching donor is not necessarily identified within a donor search.

**Figure 1:**
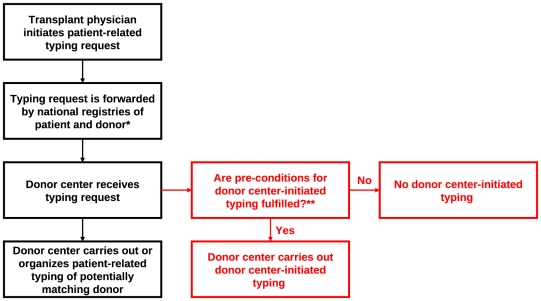
Schematic overview on the standard process of unrelated donor search (black) and the donor center-initiated typing project (red). *: Both national registries may be identical if donor and patient live in the same country. **: The donor selection process is described in the Methods section. An overview is given in Figure 2.Here, we present results of a project that combines donor center-initiated HLA typing with actual donor searches. We intended to show that this approach is suited to increase chances of patients with ongoing donor searches to find matching donors.

Available HLA phenotype data of registered donors are routinely extended either by donor center-initiated HLA typing (prospective typing), generally based on selection criteria as donor age or gender or the HLA information available so far, or by patient-related typing requests within actual donor searches. A patient-related typing request is initiated by the responsible transplant physician, forwarded by the national registries involved, and executed by the donor center that has recruited the respective donor. Figure 1 shows a schematic overview of this process.

## Methods

**Figure 2:**
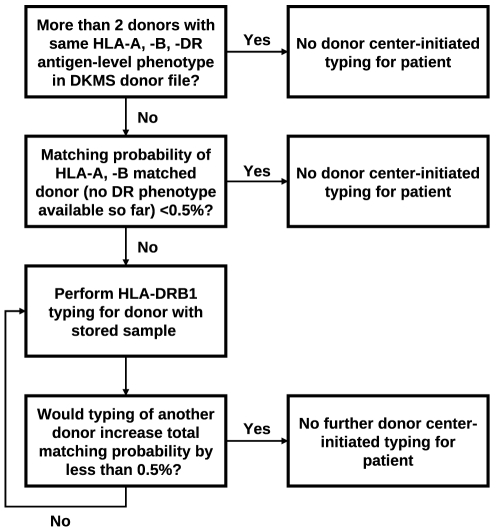
Overview on the selection process for donor center-initiated HLA-DRB1 typing.

We analyzed 9,478 donor searches between November 2009 and September 2010 that we became aware of due to patient-related HLA typing requests for donors of DKMS German Bone Marrow Donor Center. We then carried out additional donor center-initiated HLA-DRB1 typing of selected donors who were so far only typed for the HLA genes A, B and optionally C but not DRB1 (Figure 1). Donors were selected for additional typing as follows (Figure 2): Only patients with ≤2 donors on the DKMS file who matched on 3-locus (HLA-A, -B, -DR) low-resolution (antigen) level were considered in order to focus on difficult searches. We then calculated for each included patient the probability that a donor who matched on 2-locus (HLA-A, -B) low-resolution level was also a match on the 3-locus (HLA-A, -B, -DR) low-resolution level. Calculations were based on 3-locus low-resolution haplotype frequencies of the German population. Information on the HLA-C locus was – where available – not considered. Finally, donors were selected for additional HLA typing until HLA typing of one additional donor would have increased the total probability to find at least one matching donor for a specific patient by less than 0.5%. Younger donors were selected first. No donors at all were selected if the calculated individual matching probability was smaller than 0.5%. Besides, only donors for whom a stored sample for further HLA typing was available were included. Starting in February 2010, an age limit of 45 years was applied in the donor selection process.

We did not take into account potentially matching donors outside the DKMS donor file in the worldwide registry as we had no information if these donors were available, were already requested for patient-related HLA typing, or were intentionally not considered by the responsible transplant physicians.

HLA typing results of project donors were submitted to registries without references to the specific patients for whom the donors had been included in the project. Furthermore, we did not inform the responsible transplant physicians about the donor center-initiated typing efforts carried out in order to support the donor searches of their patients. This approach was chosen to avoid interferences with the standard search process. Transplant physicians became aware of the extended HLA information of project donors by routinely rechecking registry data during donor searches.

Donors who were selected for donor center-initiated typing within the project were followed-up with respect to subsequent requests for confirmatory typing (CT; a mandatory step in the donor search process that ensures the correctness of donor HLA data), donor work-up (medical donor clearing and other preparatory steps prior to donation), and stem cell donation.

At stem cell donor registration, each donor signed an informed consent form covering sample collection and storage, HLA typing of the sample, data storage and transmission of anonymous data to domestic and foreign registries. All donors who were included in the analyzed donor center-initiated typing project were informed by mail about the continuative HLA typing.

In order to avoid delays of the donor search, we initiated HLA typing and informed donors at the same time. Donors who withdrew their willingness to donate stem cells, were temporarily unavailable, or unable to donate for health-related reasons could mark respective statements on a response sheet that was attached to the mail. According to their feedback, they were temporarily or permanently excluded from the donor file. HLA typing results of these donors were included in the analysis regarding 3-locus low-resolution matched donors who were identified within the project.

No ethics committee approval was obtained as donor center-initiated typing projects are standard procedures of donor centers that are covered by the consent form signed at recruitment.

## Results

8,861 donors were contacted for additional donor center-initiated HLA-DRB1 typing within 1,089 of the 9,478 analyzed donor searches. Within 28 days after contact, 382 donors (4.3%) declared their unwillingness or lack of ability to donate stem cells or were excluded from the donor file for other reasons.

The maximum number of donors who were contacted for a specific patient as they were potential matches based on their HLA-A, -B low-resolution phenotypes was 70. For 179 patients, we identified in total 236 donors who matched on the 3-locus low-resolution level with their respective target patients.

76 confirmatory typing and 17 work-up requests were submitted for donors with donor center-initiated HLA typing, and 12 of these donors donated stem cells so far. 4 donor work-ups are currently processed, one donor was unable to donate for medical reasons. Of these requests and donations, 35 confirmatory typing requests and 10 work-up requests were made for the original target patients. 8 donors donated for their respective target patients, one donor was unable to donate, and one work-up for the target patient is currently processed.

Donations for target patients took place between 70 and 357 days (85.5 days median, 125.4 days average) after the respective donor center-initiated typing requests. Interval lengths of the various steps of the donor search process are shown in Table 1.

**Table 1:** Lengths of various intervals in the donor search process from donor center-initiated HLA typing request to stem cell donation.

t of interval	End of interval	Minimal interval length	Maximal interval length	Median interval length	Average interval length
Donor center-initiated HLA typing request	Submission of typing results to registry	3	29	14.8	17.5
Submission of typing results to registry	CT request	3	87	22.9	12.5
CT request	Provision of CT sample	8	18	10.9	11.5
Provision of CT sample	Work-up request	2	186	42.1	19.5
Work-up request	Stem cell donation	24	48	34.8	33.0
Donor center-initiated HLA typing request	Stem cell donation	70	357	125.4	85.5

Details regarding the 8 donors who donated for their target patients are displayed in Table 2. It shows that the study donors were better matches than the externally requested donors in cases 1-3 and 7. In cases 4, 5 and 8, the externally requested donors were not available for donation. In case 4, the study donor was also a better match than the externally requested donor would have been. In case 6, both the externally requested donor and the study donor were 10/10 matches. As younger donors are often preferred by transplant physicians [7], the considerably lower age of the study donor compared to the externally requested donor (27 versus 51 years) may have been essential for the final selection decision.

**Table 2:** Overview on donors who donated stem cells for their respective target patients after donor center-initiated typing.

#	Patient	Externally requested donor				Finally donating project donor			
	HLA	HLA before request	HLA	Age	Gender	HLA before donorcenter-intiated typing	HLA	Age	Gender
1	A*26:08,32:01,	A*26:XX,32:AE,	A* 26:01,32:01,	44	M	A26,32,	10/10 allele-level match	58	F
	B*08:01,39:01,	B*08:XX,39:AZRG	B*08:01,38:01,			B8,39			
	C*07:01,12:03,		C*07:01,12:03,						
	DRB1*01:01,03:01,		DRB1*01:01,03:01,						
	DQB1*02:01,05:01		DQB1*02:01,05:01						
2	A*01:01,24:02,	A*01:CNJK,01:CNJK,	A*01:01,01:01,	42	F	A*01:XX,24:XX,	10/10 allele-level match	54	M
	B*14:02,39:06,	B*14:02,39:06,	B*14:02,39:06,			B*14:BD,39:06			
	C*07:02,08:02,	C*07:CESP,08:AKZ,	C*07:02,08:02,						
	DRB1*03:01,08:01,	DRB1*03:01,08:01	DRB1*03:01,08:01,						
	DQB1*02:01,04:02		DQB1*02:01,04:02						
3	A*26:01,32:01,	A* 01:ENWD,26:GAX,	A* 01:01,26:01,	28	M	A*26:KXM,32:01,	8/8 allele-level match	27	M
	B*08:01,49:01,	B*08:XKT,49:01,	B*08:01,49:01,			B*08:XX,49:01			
	C*07:01,07:01,	C*07:CVAG,07:CVAG,	C*07:01,07:01,						
	DRB1*03:01, 07:01	DRB1*03:01, 07:01,	DRB1*03:01,07:01,						
		DQB1*02:01, 03:03	DQB1*02:01,03:03						
4	A*11:01,32:01,	A* 03:CVAB,11:BDFZ,	Donor not available	32	M	A*11:ZPJ,32:AE,	10/10 allele-level match	41	M
	B*07:02,52:01,	B*07:CZZS,52:AH,				B*07:AUSU,52:AE			
	C*07:02,12:02,	C*07:WCP,12:02,							
	DRB1*15:02,16:01,	DRB1*15:02,16:01,							
	DQB1*05:02,06:01	DQB1*05:02,06:01							
5	A*23:01,29:02,	A23,29,	Donor not available	46	F	A*23:XX,29:ASBU,	A*23:01,29:02,	44	F
	B*44:03,50:01,	B44,50,				B*44:ARXZ,50:MS	B*44:03,50:01,		
	C*04:01,06:02,						C*04:01,06:02,		
	DRB1*07:01,08:06,	DRB1*07:01,08:06					DRB1*07:01,08:06,		
	DQB1*02:02,06:02						DQB1* 03:03,06:02		
6	A*02:01,24:02,	A*02:DFKP,24:CWFP,	10/10 allele-level match	51	F	A*02:ARDA,24:AREC,	10/10 allele-level match	27	F
	B*35:02,45:01,	B*35:02,45:AH,				B*35:ND,45:01			
	C*04:01,06:02,	C*04:CVAF,06:02,							
	DRB1*04:05,11:04,	DRB1*04:CAU,11:ATF,							
	DQB1*03:01,03:02	DQB1*03:01,03:02							
7	A*03:01,26:01,	A3,26,	A3,26,	52	F	A*03:XX,26:XX,	10/10 allele-level match	42	F
	B*07:02,40:02,	B7,61	B7,61,			B*07:XX,40:CDDW			
	C*02:02,07:02,		C2,7,						
	DRB1*15:01,15:01,	DRB1*15:XX,15:XX	DR7,15						
	DQB1*06:02,06:02		DQ2,6*						
8	A*02:01,26:01,	A*02:GNF,26:01	Donor not available	23	M	A*02:XX,26:KXM,	A*02:GFFM,26:GARM,	52	M
	B*07:02,55:01,	B*07:02,55:01,				B*07:XX,55:MZ	B*07:DJJH,55:AUX,		
	C*03:03,07:02,	C*03:03,07:02,					C*03:FXFG,07:FXFR,		
	DRB1*13:01,14:01,	DRB1*13:01,14:BCAD					DRB1*13:HUJ,14:BZFS,		
	DQB1:05:03,06:03						DQB1*05:03,06:03		
							(potential 10/10 allele-level match)		

Mismatches are underlined.

*: CT result as provided by transplant center.

## Discussion

Our practical study was intended to provide a proof of principle if donor center-initiated HLA typing can improve the success chances of ongoing donor searches. This proof was successfully made.

The median and average time from donor center-initiated typing to stem cell donation (85.5 days and 125.4 days, respectively) suggest that donor-center initiated typing might often be too time-consuming to support donor searches as many stem cell transplantations are urgent. The detailed analysis of sub-intervals in Table 1, however, shows that donor-center initiated typing contributes only the minor part to the total interval from donor center-initiated typing request to stem cell donation. Besides, the average values of time interval lengths were strongly influenced by one donor search that seemed to be not time-critical (87 days from submission of results of donor center-initiated HLA typing to CT request, 186 days from provision of CT sample to work-up request). Nevertheless, it might happen in very urgent cases that the time needed for donor center-initiated typing prevents consideration of the requested donor for donation.

Eight donations resulted from donor center-initiated HLA typing of more than 8,000 donors within the study. These figures raise questions regarding the cost-benefit ratio of the analyzed HLA typing efforts. We did not carry out a formal cost-benefit analysis. Generally, cost-benefit analyses of ongoing donor recruitment or continuative HLA typing of already registered donors are complicated by several practical and ethical problems [7]–[9]. The following arguments, however, suggest that it is generally indicated to allocate resources for donor-center initiated HLA typing as support for ongoing donor searches as described in this work: First, 4 project donors have already donated stem cells for patients different from the one who caused their inclusion in the project. It is well-known that the donation probability of registered stem cell donors depends on the completeness of their HLA typing results [7], [10], [11]. It is, therefore, highly probable that further additional donations will result from the project in the future. Second, continuative donor center-initiated HLA typing of already registered donors is carried out routinely by many donor centers. Respective programs often include donor age or gender as donor selection criteria. Here lies, apart from the focus on specific patients, the main difference to our study that also included older donors and did not consider donor gender. It is, however, possible to improve the cost-benefit ratio of patient-focused donor center-initiated typing projects by inclusion of respective criteria. This is why we introduced an age limit of 45 years. Third, the cost-benefit ratio should be further increasable by consideration of 4-locus high-resolution instead of 3-locus low-resolution matching probabilities in the donor selection process.

The 8 donating study donors who were obviously – after additional donor center-initiated HLA typing, confirmatory typing and medical clearing – assessed as best available donors for their target patients by the responsible transplant physicians had originally not been requested for additional typing based on their available HLA information that did not include HLA-DRB1. It is, therefore, doubtful if they had advanced to donation without donor center-initiated HLA typing.

This finding raises the question if there are deficiencies of the existing donor search process regarding the use of incompletely typed donors, especially of donors who are typed for HLA-A and -B only. An infrequent use of donors with this typing profile has been reported before [10]. Deficiencies of the search process would also be consistent with observed discrepancies between calculated [12] and reported [13], [14] probabilities to find completely matched donors for Caucasian patients.

The restriction to 3-locus low-resolution matching in the donor selection process and the non-availability of data on patient-related requests for non-DKMS donors within the considered donor searches are the major limitations of the study. It is, therefore, not possible to draw definite conclusions regarding the efficiency of the donor search process from our results. Due to the high relevance of this issue for optimal donor selection and thus for the best possible patient care, further analyses of this question are urgently required.

It is obvious that full HLA typing at donor recruitment reduces the probability of non-finding the optimal stem cell donor. As new donors are increasingly typed more completely due to advances in typing technology and related cost reductions, the probability of non-finding the optimal donor should decrease in the future. However, there will be millions of partially typed donors in the worldwide donor file for many years to come. There is a need for strategies that make sure that these donors are utilized properly in actual donor searches. Haplotype frequency-based search algorithms [15], [16] will probably play a major role in this effort.

Many donor centers regularly run prospective typing projects in order to increase the completeness of HLA information of their registered donors. Our results suggest it might be indicated to use the funds available for such projects at least partly for donor center-initiated HLA typing as support for currently ongoing donor searches as described in this work. We plan, therefore, to continue with this project with a refined donor selection process that is based on probabilities for 4-locus high-resolution instead of 3-locus low-resolution matching.
